# Confounding giant right atrial mass

**DOI:** 10.1186/s43044-024-00533-y

**Published:** 2024-08-18

**Authors:** Sefa Tatar, Ahmet Taha Sahin, Mehmet Işık, Niyazi Görmüş

**Affiliations:** 1 Department of Cardiology, Meram Faculty of Medicine, Necmettin Erbakan University, Konya, Turkey; 2 Department of Cardiovasculer Surgery, Meram Faculty of Medicine Necmettin, Erbakan University, Konya, Turkey

**Keywords:** Cardiac tumors, Hydatid cyst, Atrial myxomas, Surgery

## Abstract

**Background:**

Primary cardiac tumors are uncommon, with approximately 70–80% classified as benign. Myxomas constitute roughly half of all benign cardiac tumors, while cardiac hydatid cysts are exceptionally rare. Shortness of breath is a prominent symptom associated with these conditions. Echocardiography serves as the primary diagnostic tool, enabling early detection. The preferred course of action involves the surgical excision of the mass.

**Case presentation:**

Our first case, a 51-year-old female presented to the cardiology outpatient clinic with complaints of shortness of breath and palpitations persisting for 3 months. Physical examination and tests were within normal limits. Echocardiography revealed a right atrial myxoma measuring 65 * 35 mm. Despite not affecting valve and ventricular functions, the mass was surgically resected. Our second case, a 55-year-old male admitted to the cardiology clinic with recurrent fever, shortness of breath, and chest pain. Physical examination and tests were normal. Echocardiography revealed an echogenic mass with a septate appearance adhering to the free wall of the right atrium. The patient, diagnosed with a hydatid cyst, underwent surgical resection.

**Conclusions:**

We came across two different cases of RA masses which happened to be myxoma and hydatid cysts, and we managed accordingly. The right atrial myxomas typically present with nonspecific shortness of breath unless they cause valve obstruction. In cases where clinical findings suggest infection in the right atrial masses, it is essential to consider hydatid cyst as a potential diagnosis alongside the initial consideration of vegetation.

## Background

Primary cardiac tumors are exceedingly rare, with approximately 70–80% of cases identified as benign. Myxomas, which represent around half of all benign cardiac tumors, are typically found within the atria, with the left atrium being the most common location [1]. Although infrequent, instances of myxoma localization in the right atrium have also been documented [2]. Clinical manifestations vary depending on the size and specific location of the mass, with shortness of breath being a predominant symptom [3]. Structures like these cannot always be clearly distinguished as myxoma or other masses through echocardiography or other imaging methods. This can lead to misdiagnosis and delayed treatment. This article aims to present two cases featuring giant right atrial masses, both associated with complaints of shortness of breath.

## Case presentation

**Fig. 1:**
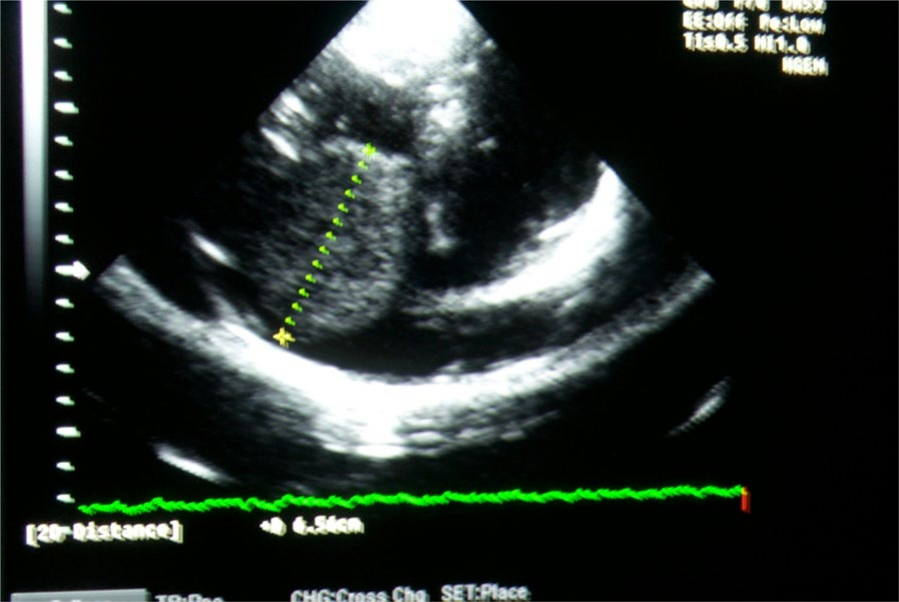
Appearance of the right atrial mass on transthoracic echocardiography

**Fig. 2:**
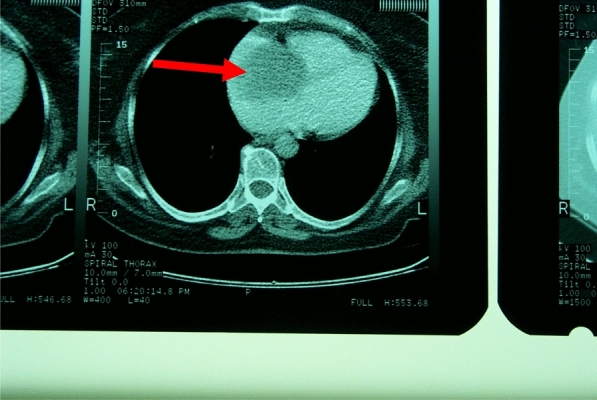
Appearance of the right atrial mass on computed tomography

**Fig. 3:**
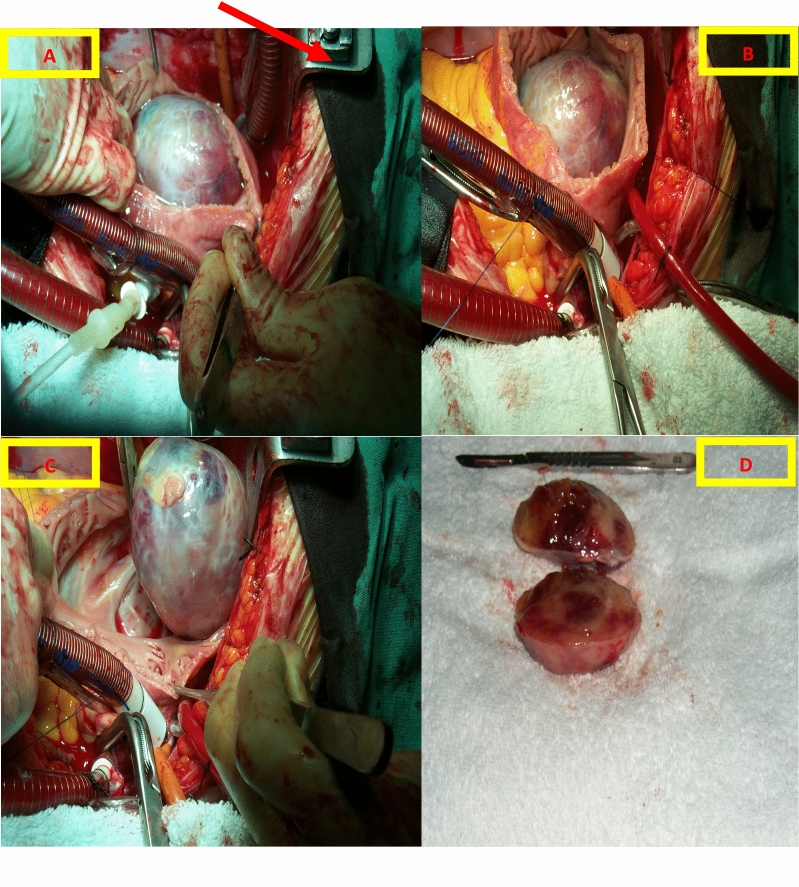
Intraoperative appearance of the right atrial mass

Our initial case involves a 51-year-old female patient with no known history of systemic or cardiac diseases. She presented to the cardiology outpatient clinic with complaints of nonspecific shortness of breath and palpitations. According to her medical history, the symptoms had been intermittent for the past 3 months, escalating in frequency over the past month. The patient reported no family history of cardiac issues and was not on regular medication. Upon physical examination, rhythmic heart sounds were noted, and no distinct murmurs were observed. The respiratory system examination revealed normal findings, with no pathology detected in other systemic assessments. Electrocardiography (ECG) revealed no ischemic or pathological changes in sinus rhythm. Laboratory examinations showed no abnormalities. Transthoracic echocardiography (TTE) revealed a mass measuring 65 * 35 mm in diameter within the right atrium (Fig. 1). The patient's tricuspid valve and right ventricular functions appeared normal. Consequently, a surgical intervention was deemed necessary. Prior to surgery, coronary angiography was conducted, revealing no obstructive stenosis in the coronary arteries. During the surgical procedure, it was observed that the mass did not invade surrounding tissues and was attached to the free wall of the right atrium via a peduncle (Figs. 2 and 3). Subsequent to the surgical intervention, pathological examinations revealed the presence of a myxoma. The patient experienced an uneventful postoperative period and was subsequently discharged.

**Fig. 4:**
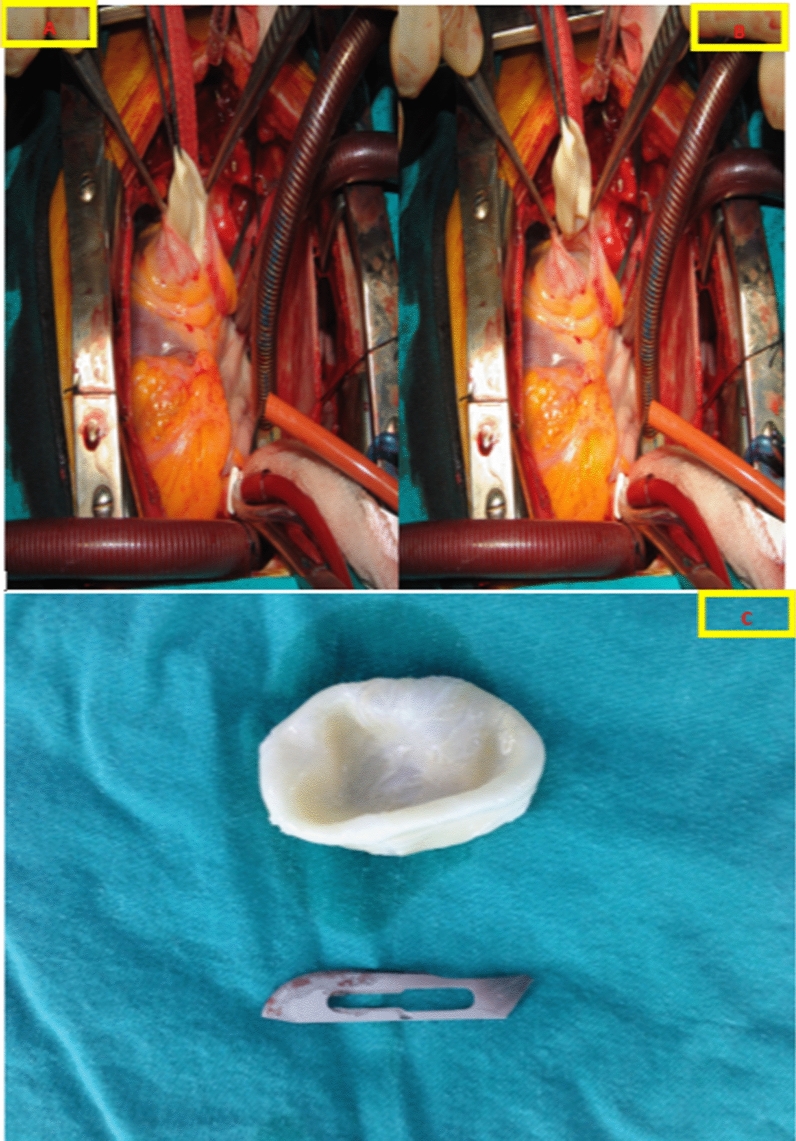
Intraoperative appearance of the right atrial hydatid cyst

Our second case involves a 55-year-old male patient with a documented history of hypertension. He presented to the cardiology outpatient clinic complaining of recurrent fever, shortness of breath, and chest pain, with symptoms persisting for the past year. The patient was engaged in animal husbandry and was under calcium channel blocker medication for hypertension. No evident abnormalities were noted in the electrocardiogram (ECG). Transthoracic echocardiography (TTE) unveiled an echogenic mass measuring 55 * 30 mm, exhibiting a septate appearance, and being attached to the free wall of the right atrium. Laboratory parameters indicated elevated infectious markers. The patient's tricuspid valve and right ventricular functions exhibited no pathological findings. Consequently, a decision for surgical intervention was made. Prior to surgery, coronary angiography revealed no obstructive stenosis in the coronary arteries. Intraoperatively, the mass was identified as white, septate, and filled with gelatinous fluid (Fig. 4). The mass, initially suspected to be a hydatid cyst, was aspirated and emptied during the procedure. Subsequent pathological examination confirmed the diagnosis of a hydatid cyst, and the mass was successfully removed in its entirety. The patient received metronidazole treatment for 10 days and was subsequently discharged.

## Discussion

Our article holds significance due to the rarity of the right atrial masses. Particularly, large atrial masses like these may be confused with each other until the intraoperative process due to nonspecific symptoms and lack of specific findings in imaging methods. Imaging methods such as transthoracic echocardiography and computed tomography are crucial diagnostic tools for assessing the size, location, and relationship of the mass with surrounding tissues. However, these methods do not always allow for a definitive diagnosis. Some masses may appear similar to myxoma in imaging tests but turn out to be metastatic malignant tumors. In some cases, thrombus formation may mimic myxoma. Differentiating these structures before surgery is important, but some cases can only be distinguished during surgery. In the first case, the considerable size of the mass is noteworthy as it does not elicit specific symptoms in the patient and does not impact heart functions on ECG and TTE. However, it is crucial to recognize that such sizable masses can impair tricuspid valve functions, induce dysfunction in the right ventricle, result in tumor embolization, and even lead to sudden death. Conversely, the second case is noteworthy for presenting a rare cardiac manifestation of a hydatid cyst. Upon reviewing the literature, few articles discuss the association between hydatid cysts and the heart. In this instance, the preservation of right heart functions despite the mass effect, the absence of sepsis development despite an ongoing infectious situation, and the prevention of cyst rupture, especially given its substantial size, are significant observations. Both cases were fortunate not to result in pulmonary embolism due to tumor embolization. It is pertinent to mention that myxomas represent the most common primary tumors in the heart, with approximately 15–20% located in the right atrium [4]. Typically found in the fossa ovalis region of the right atrium, myxomas very rarely attach to the atrium wall [5].

In the differential diagnosis of the right atrial masses, consideration should be given to various structures, including thrombus, myxoma, vegetation, tumor metastasis, interatrial septal pathologies, eustachian valve, and the Chiari network [6, 7]. Thrombus typically exhibits mobility and a lobulated character, with irregular edges. In contrast, myxomas are relatively fixed, characterized by smooth edges, and present as solid masses. The right atrial myxomas tend to be larger with a broader base. The most concerning complication associated with myxomas is their potential to obstruct the tricuspid valve. However, in our first case, despite the substantial size of the mass, it did not lead to valve obstruction or insufficiency. This is likely attributed to the fact that the mass was not attached to the interatrial septum but rather to the free wall of the right atrium.

In our second case, the fragmented structure of the mass, elevated infectious parameters, and the presence of fever were indicative of a vegetation. These findings highlight the importance of a comprehensive approach to differential diagnosis, considering both clinical and imaging features to accurately identify the nature of the right atrial masses. The consideration of vegetation persisted until the intraoperative phase, shaping the treatment plan accordingly. However, upon macroscopic examination, it became evident that the mass was, in fact, a hydatid cyst. Furthermore, the absence of the left ventricular wall motion defects, ischemic heart disease, any underlying clinical conditions predisposing to thrombogenicity, and the normal dimensions of the heart chambers collectively steered us away from the initial diagnosis of a thrombus.

Cardiac hydatid cysts are exceptionally rare, constituting approximately 1% of all hydatid cyst cases [8]. They are commonly located in the ventricles and interventricular septum, with the right atrial and pericardial involvement being extremely uncommon. Despite the diagnostic challenges, modalities such as transthoracic echocardiography (TTE), transesophageal echocardiography, cardiac computed tomography, and cardiac magnetic resonance imaging prove valuable in achieving a diagnosis. Surgical intervention stands as the definitive treatment, and postoperatively, the recurrence rate can be mitigated with medications such as albendazole and metronidazole. The rupture of a cyst bears the risk of disseminating the disease, potentially leading to sepsis. Macroscopically, myxomas can manifest in two distinct forms: the globular type, characterized by a round and smooth surface, and the polypoid type, featuring an irregular surface [9]. Additionally, myxomas are categorized into sporadic and familial types. The common sporadic type typically exhibits a typical location, while the rare familial type tends to present atypically, often in younger individuals, and may involve multiple structures [10]. Clinical manifestations and symptoms vary depending on the mass's location. Myxomas causing obstruction and insufficiency in the atrioventricular valve typically lead to complaints such as orthopnea, dyspnea, cough, hemoptysis, and rhythm disorders. In our case, the symptoms were not pronounced as the mass had minimal impact on the valves and right ventricular functions.

The left-sided myxomas may result in cerebrovascular disease, mesenteric ischemia, and renal infarction due to peripheral and systemic embolization. Additionally, nonspecific systemic symptoms such as fever, fatigue, weight loss, and joint pain may also be observed. Echocardiography holds a crucial role in the differential diagnosis of the right atrial masses. In cases where echocardiography proves insufficient, transesophageal echocardiography, cardiac computed tomography, and cardiac magnetic resonance imaging are valuable adjuncts. The standard treatment for myxomas is surgical excision. Patients inadequately resected, those with multiple structures, familial types, or those developing as tumor metastases have a higher recurrence rate [11]. Surgery should be promptly scheduled upon diagnosis due to potential complications such as acute valve obstruction, pulmonary embolism, and sepsis.

## Conclusions

In conclusion, in cases where clinical findings suggest infection in the right atrial masses, it is essential to consider hydatid cyst as a potential diagnosis, alongside the initial consideration of vegetation. Additionally, it is noteworthy that the right atrial myxomas may manifest with nonspecific shortness of breath unless they lead to valve obstruction. A comprehensive diagnostic approach, incorporating imaging techniques such as echocardiography, is crucial for accurate and timely identification of the underlying pathology in the right atrial masses.

## Data Availability

All data generated or analyzed during this study are included in this published article. Authors’ contributions All authors read and approved the final version of the manuscript.

## Abbreviations

ECG: Electrocardiography
TTE: Transthoracic echocardiography

## References

[1] Parato VM, Nocco S, Alunni G, Francesco B, Serenella C, Umberto C (2023) Imaging of cardiac masses: an updated overview. J Cardiovasc Echogr 32(2):65–75 10.4103/jcecho.jcecho_18_22 PMC9558634 36249434

[2] Liu Y, Li X, Liu Z, Jiang Y, Lu C, Ge S et al (2023) Clinical characteristics and surgical outcomes of right heart myxoma and its comparison with left heart myxoma: a single-center retrospective study. Anatol J Cardiol 27(3):146–152 36856593 10.14744/AnatolJCardiol.2022.2585 PMC9995553

[3] Li H, Guo H, Xiong H, Xu J, Wang W, Hu S (2016) Clinical features and surgical results of right atrial myxoma. J Card Surg 31(1):15–17 26585438 10.1111/jocs.12663

[4] Meng Q, Lai H, Lima J, Tong W, Qian Y, Lai S (2002) Echocardiographic and pathologic characteristics of primary cardiac tumors: a study of 149 cases. Int J Cardiol 84:69–75 12104067 10.1016/S0167-5273(02)00136-5

[5] Peters PJ, Reinhardt S (2006) The echocardiographic evaluation of intracardiac masses: a review. J Am Soc Echocardiogr 19:230–240 16455432 10.1016/j.echo.2005.10.015

[6] Harrity PJ, Tazelear HD, Edwards WD, Orszulak TA, Freeman WK (1995) Intracardiac varices of the right atrium: a case report and review of the literature. Int J Cardiol 48:177–181 7774997 10.1016/0167-5273(94)02232-8

[7] Panthee N, Koirala R, Rajbhandari N, Pradhan S (2020) Thrombus straddling patent foramen ovale and massive pulmonary embolism. Indian J Thorac Cardiovasc Surg 36(6):635–638 33100625 10.1007/s12055-020-01003-1 PMC7572990

[8] Hidatik K (2005) Pulmoner tutulumla beraber tekrarlayan kardiyak kist hidatik. Turkish J Thorac Cardiovasc Surg 13:59–61

[9] Ha JW, Kang WC, Chung N, Chang BC, Rim SJ, Kwon JW et al (1999) Echocardiographic and morphologic characteristics of left atrial myxoma and their relation to systemic embolism. Am J Cardiol 83:1579–1582 10363879 10.1016/S0002-9149(99)00156-3

[10] Roberts WC (1997) Primary and secondary neoplasms of the heart. Am J Cardiol 80:671–682 9295010 10.1016/S0002-9149(97)00587-0

[11] Kaplan M, Demirtaş MM, Çimen S, Gerçekoğlu H, Yapıcı F, Özler A (2002) Cardiac myxomas: surgical experience with 45 cases. Turk J Thorac Cardiovasc Surg 10:11–14

